# Significance of Lymph Node Metastasis in the Treatment of Gastric Cancer and Current Challenges in Determining the Extent of Metastasis

**DOI:** 10.3389/fonc.2021.806162

**Published:** 2022-01-07

**Authors:** Shinichi Kinami, Hitoshi Saito, Hiroyuki Takamura

**Affiliations:** ^1^ Department of Surgical Oncology, Kanazawa Medical University, 1-1 Daigaku, Uchinada-machi, Kahoku-gun, Japan; ^2^ Department of General and Gastroenterologic Surgery, Kanazawa Medical University Himi Municipal Hospital, Himi City, Japan

**Keywords:** gastric cancer, lymph node metastasis, MDCT, sentinel node, staging

## Abstract

The stomach exhibits abundant lymphatic flow, and metastasis to lymph nodes is common. In the case of gastric cancer, there is a regularity to the spread of lymph node metastasis, and it does not easily metastasize outside the regional nodes. Furthermore, when its extent is limited, nodal metastasis of gastric cancer can be cured by appropriate lymph node dissection. Therefore, identifying and determining the extent of lymph node metastasis is important for ensuring accurate diagnosis and appropriate surgical treatment in patients with gastric cancer. However, precise detection of lymph node metastasis remains difficult. Most nodal metastases in gastric cancer are microscopic metastases, which often occur in small-sized lymph nodes, and are thus difficult to diagnose both preoperatively and intraoperatively. Preoperative nodal diagnoses are mainly made using computed tomography, although the specificity of this method is low because it is mainly based on the size of the lymph node. Furthermore, peripheral nodal metastases cannot be palpated intraoperatively, nodal harvesting of resected specimens remains difficult, and the number of lymph nodes detected vary greatly depending on the skill of the technician. Based on these findings, gastrectomy with prophylactic lymph node dissection is considered the standard surgical procedure for gastric cancer. In contrast, several groups have examined the value of sentinel node biopsy for accurately evaluating nodal metastasis in patients with early gastric cancer, reporting high sensitivity and accuracy. Sentinel node biopsy is also important for individualizing and optimizing the extent of uniform prophylactic lymph node dissection and determining whether patients are indicated for function-preserving curative gastrectomy, which is superior in preventing post-gastrectomy symptoms and maintaining dietary habits. Notably, advancements in surgical treatment for early gastric cancer are expected to result in individualized surgical strategies with sentinel node biopsy. Chemotherapy for advanced gastric cancer has also progressed, and conversion gastrectomy can now be performed after downstaging, even in cases previously regarded as inoperable. In this review, we discuss the importance of determining lymph node metastasis in the treatment of gastric cancer, the associated difficulties, and the need to investigate strategies that can improve the diagnosis of lymph node metastasis.

## Introduction

Lymph node metastasis is an important determinant of disease progression in patients with gastric cancer. The stage of gastric cancer without distant metastasis is determined based on the depth of invasion and the degree of lymph node metastasis (1–3). In recent years, determining the degree of lymph node metastasis after gastric cancer surgery has become essential for selecting appropriate adjuvant therapy and improving prognosis, as the effect of adjuvant chemotherapy in suppressing recurrence has become clear (4–8). In addition, given that it can be cured to some extent by prophylactic lymph node dissection, preoperative diagnosis of lymph node metastasis is an important factor when planning surgical treatment for patients with gastric cancer (9–11). For these reasons, numerous studies have investigated the diagnosis, pathophysiology, and treatment of lymph node metastasis in the context of gastric cancer (12–14). However, diagnosis of nodal metastasis remains difficult in these patients, and several problems with detection remain unresolved. In this review, we discuss the importance of determining lymph node metastasis in the treatment of gastric cancer and the associated difficulties.

## Characteristics of Lymph Node Metastasis in Patients With Gastric Cancer

Lymph node metastasis is a common form of metastasis in patients with gastric cancer (9). Cancer that invades within the submucosa is defined as early gastric cancer, regardless of the presence or absence of metastasis (15). The rate of hematogenous metastasis in patients with early gastric cancer is approximately 0.2% (16), and peritoneal metastasis is unlikely to occur (17), whereas the incidence of lymph node metastasis is approximately 10% (18). The relatively high rate of lymph node metastasis in patients with gastric cancer can be attributed to the abundant lymphatic flow in the stomach (19, 20). Indeed, there is a rich lymphatic network in the submucosa, and immunohistological staining using D2-40 have revealed that there are abundant lymphatic vessels near the muscularis mucosae (21). This physiological environment explains the frequency of lymph node metastasis even in cases of early gastric cancer (19, 21).

The incidence of lymph node metastasis in gastric cancer is closely related to the depth of invasion (17). According to the database of the Cancer Institute Hospital of the Japan Foundation for Cancer Research, which is considered the most reliable large-scale database, the nodal metastasis rates according to pathological depth of invasion are 2.3% for mucosal, 21.9% for submucosal, 64.2% for proper muscle-subserosal, and 86.6% for serosal exposure cancers (17).

**Figure 1:**
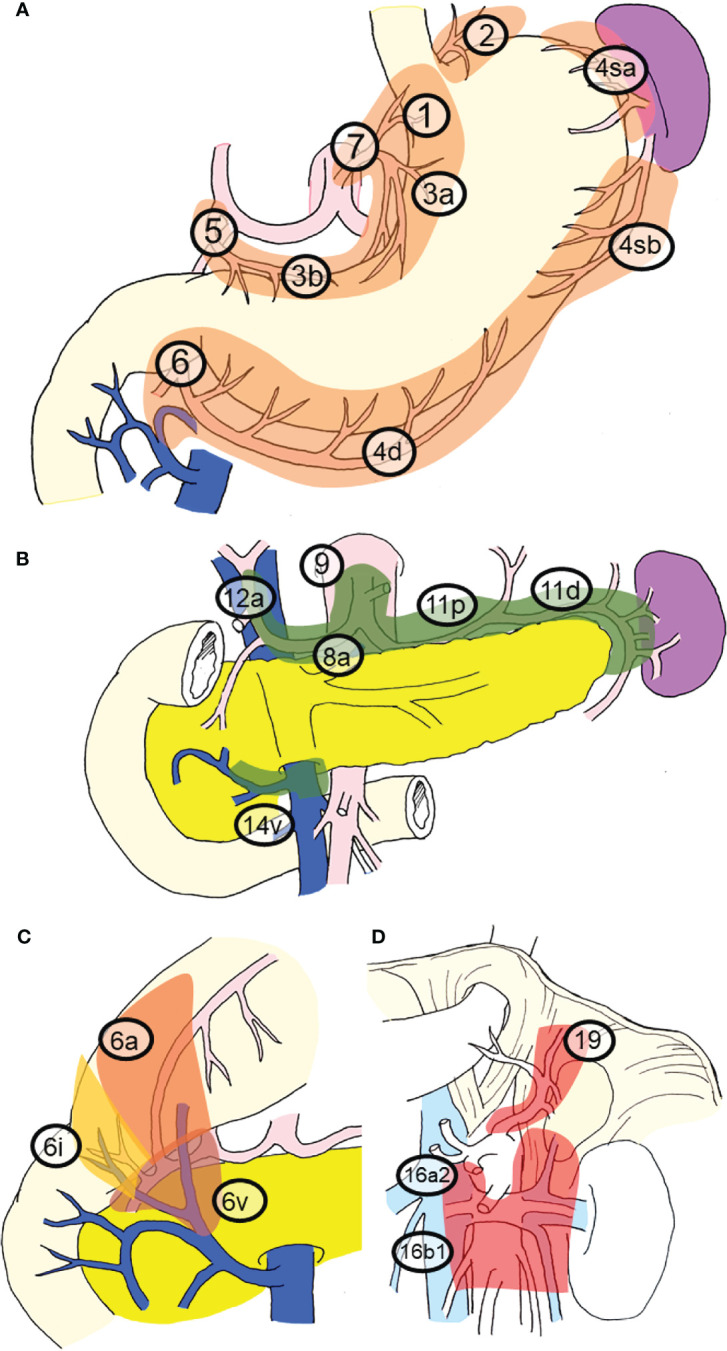
Regional lymph nodes and station numbers in gastric cancer. This classification and the station numbers are based on the Japanese Classification of Gastric Carcinoma. The details of the nodal station numbers have been described in **Table 2**. **(A)** Perigastric nodes and nodes along the left gastric artery. These nodes nearly correspond to group 1 nodes. **(B)** Nodes around the celiac artery, along the proper hepatic artery and suprapancreatic nodes. These nodes nearly correspond to the group 2 nodes. **(C)** The subcategory of No. 6 nodes. **(D)** Nodes in deeper layers. Para-aortic lymph nodes and No. 19 nodes. These nodes nearly correspond to group 3 nodes.

**Figure 2:**
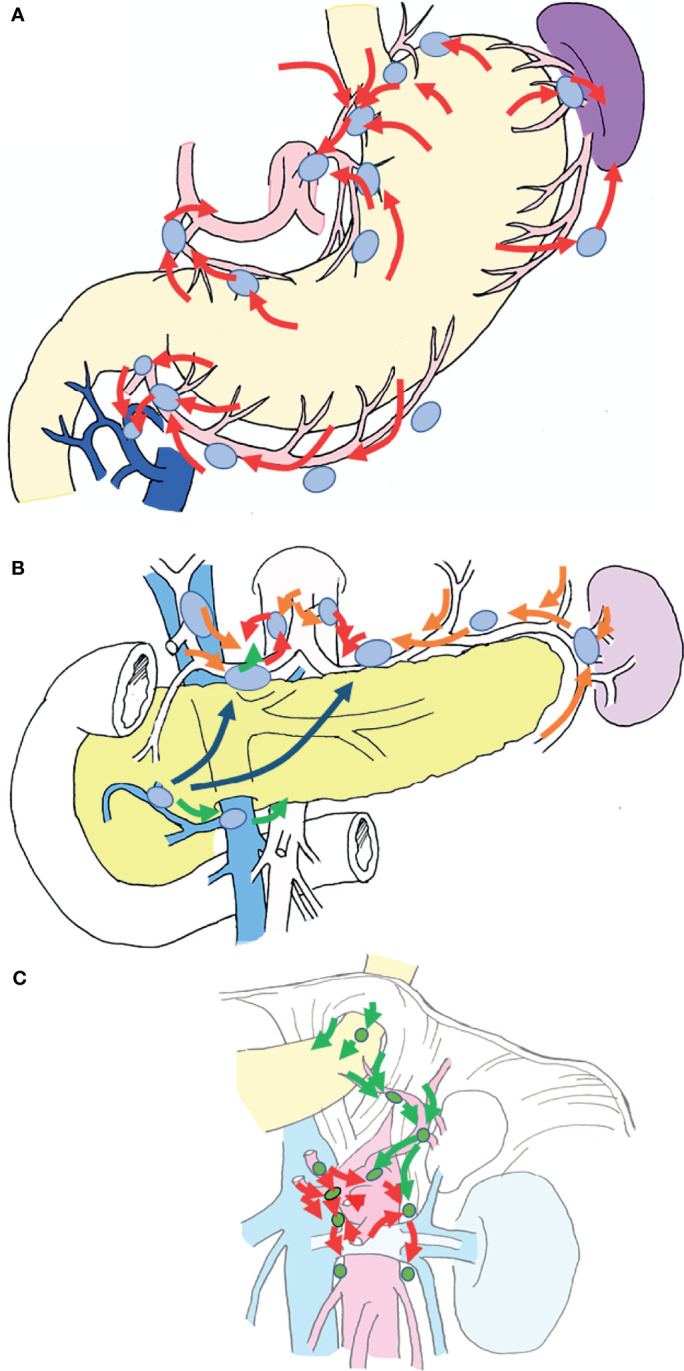
Schematic diagram of the lymphatic system of the stomach. The lymphatic flow of the stomach spreads from the perigastric nodes, *via* the suprapancreatic nodes and nodes around the celiac artery, to the para-aortic nodes, following which it enters the systemic circulation. **(A)** Lymphatic flow from the gastric wall is directed to the root of each artery *via* nearby perigastric nodes (red arrows). **(B)** The lymphatic flow into the root of each artery flows *via* suprapancreatic nodes (orange arrows) and out to the paraaortic nodes from the left and right of the celiac artery (red arrows). There are also routes from #8a to #8p (the posterior side of the common hepatic artery), and routes from #6 to the root of the superior mesenteric artery *via* #14v (green arrows). Routes from #6 to the suprapancreatic nodes *via* the lymphatics under the pancreatic capsule are also available (indigo arrows). **(C)** Lymphatic flow around the celiac artery and the superior mesenteric artery lead to the paraaortic nodes, which are the terminal lymph nodes of gastric cancer (red arrows). A route from the left dorsal side of the cardia to #16a2 lateral nodes *via* #19, along the left subphrenic artery also exists (green arrows).

Previously, pioneers in gastric cancer surgery in Japan classified regional lymph nodes along the arteries, examined lymphatic flow using tracers, and tabulated the sites of lymph node metastasis (2, 3), revealing that there is a regularity in the spread of lymph node metastases in patients with gastric cancer. **Table 1** shows that the rate of lymph node metastasis of early gastric cancer varies considerably according to station number (17, 22), based on data from representative articles. It can be seen that the lymph node metastasis rate varies considerably with the station number. Lymph nodes and lymphatic system of gastric cancer exhibit a stratified structure, consisting of three layers: the perigastric nodes and nodes along the left gastric artery; the nodes around the celiac artery, along the proper hepatic artery, and the suprapancreatic nodes; and the deeper para-aortic lymph nodes (**Figure 1**). The station numbers and definitions of the lymph nodes, which are important for gastric cancer staging and surgical treatment are precisely stated in **Table 2**. These nodes approximately correspond to group 1, 2, and 3 lymph nodes in the old Japanese Classification of Gastric Carcinoma, respectively (23). Most nodal metastases in early gastric cancer are confined to group 1 nodes, the rate of metastasis to group 2 nodes is low, and metastasis to group 3 nodes is rare. Lymph node metastases are thought to spread along with the lymphatic flow from group 1 to group 2 and then to group 3. In other words, lymph node metastasis of gastric cancer spreads from the perigastric nodes, *via* the suprapancreatic nodes and nodes around the celiac artery, to the para-aortic nodes, following which it flows out to the systemic circulation (**Figure 2**). Currently, the regional lymph nodes for gastric cancer are defined as No. 1 to No. 12 and No. 14v, and other nodal metastases are considered distant metastases (2, 3).

**Table 1:** The precise incidence of nodal metastasis of early gastric cancer in previous studies with large number of cases.

		Nakajima (17)	Tanaka (22)
**Total no. of cases**		3630	2368
**Perigastric nodes**	#1	0.90%	0.97%
	#2	0.11%	0.08%
	#3	5.9%	4.6%
	#4	3.9%	3.2%
	#5	0.47%	0.51%
	#6	3.4%	2.4%
**Nodes along left gastric artery**	#7	1.1%	1.4%
**Suprapancreatic nodes**	#8a	1.1%	0.63%
	#9	1.1%	0.72%
	#11p	0.36%	0.42%
	#11d	0.03%	0.00%
**Nodes at the hilum of spleen**	#10	0.08%	0.00%
**Nodes along proper hepatic artery**	#12a	0.06%	0.00%
**Para-aortic nodes**	#16	0.25%	0.00%

The percentage of each column represents the metastatic ratio per patients of the lymph nodes in each station.

**Table 2:** The station numbers and the definitions of the lymph nodes which are important for gastric cancer staging and surgical treatment.

Perigastric nodes	
**#1**	Nodes at the left side of cardia, including those along the first branch of the ascending limb of the left gastric artery
**#2**	Nodes at the right side of cardia, including those along the esophagocardiac branch of the left subphrenic artery
**#3a**	Nodes at the lesser curvature of stomach along the branches of the left gastric artery
**#3b**	Nodes at the lesser curvature of stomach along the second branch and distal part of the right gastric artery
**#4sa**	Nodes along the short gastric arteries
**#4sb**	Nodes at the left side of greater curvature along the left gastroepiploic artery
**#4d**	Nodes at the right side of greater curvature along the second branch and distal part of the right gastroepiploic artery
**#5**	Suprapyloric nodes along the first branch and proximal part of the right gastric artery
**#6a**	Infrapyloric nodes along the first branch and proximal part of the right gastroepiploic artery
**#6v**	Nodes along the confluence of the right gastroepiploic vein
**#6i**	Nodes along the infrapyloric artery and vein
**Nodes along the left gastric artery**	
**#7**	Nodes along the trunk of the left gastric artery between its root and the ascending branch
**Suprapancreatic nodes**	
**#8a**	Nodes at the anterosuperior side of the common hepatic artery
**#9**	Nodes around the celiac artery
**#11p**	Nodes at the proximal half side along the splenic artery
**#11d**	Nodes at the distal half side along the splenic artery
**Others of the regional lymph nodes**	
**#12a**	Nodes along the proper hepatic artery (left side nodes of the hepatoduodenal ligament)
**#14v**	Nodes along the superior mesenteric vein
**Para-aortic nodes (PAN)**	
**#16a2 lateral**	Left side of para-aortic nodes between the upper margin of the origin of the celiac artery and the lower border of the left renal vein
**#16a2 inter**	Right side of para-aortic nodes between the upper margin of the origin of the celiac artery and the lower border of the left renal vein
**#16b1 lateral**	Left side of para-aortic nodes between the lower border of the left renal vein and the upper border of the origin of the inferior mesenteric artery
**#16b1 inter**	Right side of para-aortic nodes between the lower border of the left renal vein and the upper border of the origin of the inferior mesenteric artery
**Other important nodes**	
**#8p**	Nodes at the posterior side of the common hepatic artery
**#13**	Nodes on the posterior surface of the pancreas head cranial to the duodenal papilla
**#19**	Nodes along the left subphrenic artery
**#20**	Paraesophageal nodes at the diaphragmatic esophageal hiatus

In many cancers, lymph node metastasis is an important surrogate marker of survival prognosis and an important factor when considering postoperative adjuvant therapy. The prognosis of gastric cancer also worsens as the number of lymph node metastases increases (24–27). A previous study reported that survival curves clearly deviate according to the number of lymph node metastases, based on analysis of data in the registry of the Japanese Society of Gastric Cancer, a high-quality dataset that includes information for half of all patients undergoing gastric cancer surgery in Japan (9).

The Z0011 study demonstrated that additional axillary nodal dissection after sentinel node biopsy does not improve prognosis in patients with breast cancer (28–30). Similarly, in many carcinomas, lymph node metastasis is thought to be important for accurate staging, but prophylactic nodal dissection does not improve prognosis (31–35). In contrast, the therapeutic effect of prophylactic lymph node dissection in patients with gastric cancer has been verified. The rate of nodal metastasis for early gastric cancer is approximately 10% (18), but the 5-year survival rate after gastrectomy with prophylactic nodal dissection is as high as 98% (9–11). In cases of advanced gastric cancer, D2 (nodal dissection up to group 2 nodes) is associated with better prognosis than D1 (dissection of perigastric nodes only) (36, 37). This phenomenon seems to be a unique feature of gastric cancer. It is presumed that the major difference between gastric cancer and breast cancer is related to the anatomical location of the lymph nodes. Breast cancer that has metastasized to the axillary lymph nodes can easily develop systemic metastases from these nodes. In contrast, the lymphatic system of gastric cancer exhibits a stratified structure (**Figures 1**, **2**), and there exists a state in which metastatic foci are localized around the stomach and not spread throughout the body, which is presumed to account for the prophylactic effect of lymph node dissection.

**Figure 3:**
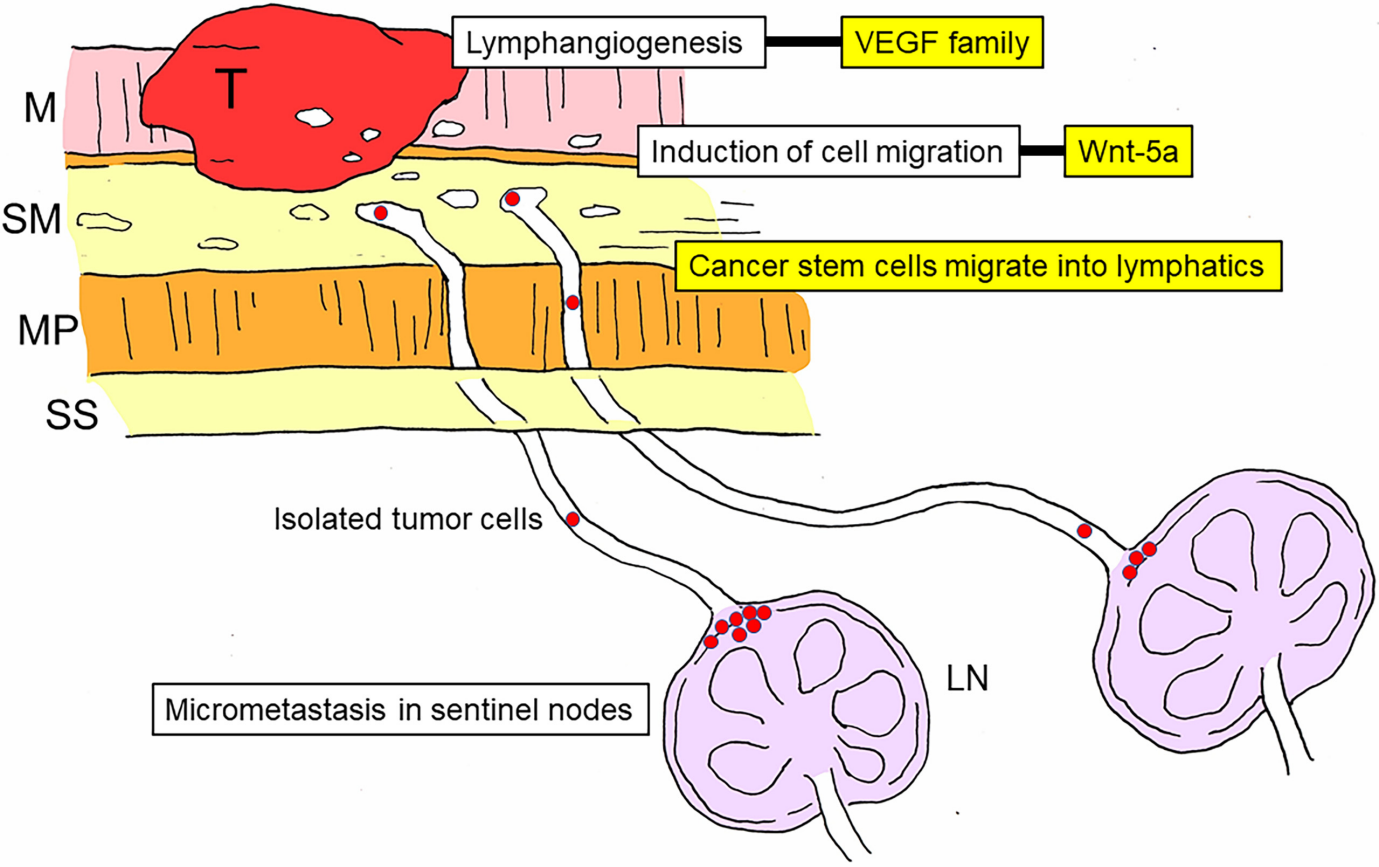
Schematic diagram of the development and molecular mechanisms of nodal metastasis in gastric cancer. Lymph node metastasis can be divided into multiple stages: lymphangiogenesis, induction of cell migration, invasion of cancer stem cells into the lymphatic system, arrival of cancer stem cells in sentinel lymph nodes, and establishment of micrometastasis in the marginal sinus. The vascular endothelial growth factor (VEGF) family is involved in lymphangiogenesis, and Wnt-5a is involved in the induction of cell migration. T, tumor; M, mucosal layer; SM, submucosal layer; MP, proper muscle layer; SS, subserosal layer; LN, lymph node; VEGF, vascular endothelial growth factor.

Previous studies have elucidated the molecular mechanisms involved in lymph node metastasis (38–41). Among them, the initial and most important process is lymphangiogenesis (42–44), which is regulated by members of the vascular endothelial growth factor (VEGF) family and their receptors (43–46). Cell migration is another important process in nodal metastasis, and Wnt-5a is thought to be among the cell migration-associated molecules involved in gastric cancer (47, 48). In addition, it is well accepted that cancer stem cells play a significant role in nodal metastasis of gastric cancer (48–50) (**Figure 3**).

## Standard Lymph Node Dissection Strategy for Gastric Cancer

Lymph node dissection for gastric cancer was well studied in Japan during the 20th century. Following the studies related to the frequency of lymph node metastasis and the prognosis of nodal dissection, the results were consolidated by the Japanese Research Society for Gastric Cancer, which revised the classification of station numbers and proposed extent of nodal dissection. In the past, the extent of nodal dissection was determined based on the location of gastric cancer (23), and strategies were based on the results of retrospective studies. The importance of prospective studies is now recognized in the field of surgical treatment. Since lymph node dissection allows for an accurate pathological diagnosis of nodal metastasis, leading to so-called stage migration (51–53), retrospective studies may overestimate the effect of lymph node dissection. Thus, the actual outcome of nodal dissection can only be evaluated in prospective studies.

Two prospective clinical trials were conducted to determine the influence of the extent of nodal dissection in gastric cancer surgery. One is JCOG9501 trial (53–56). At that time, approximately 10% of patients with para-aortic nodal metastasis survived for 5 years if these nodes were dissected (51). To verify the efficacy of para-aortic nodal dissection, a prospective randomized clinical trial was conducted, in which patients with advanced gastric cancer were randomly assigned to two treatment groups: the study group with lymph node dissection up to D2 plus para-aortic lymph node (PAN) dissection and the control group with nodal dissection up to D2 only. The results indicated that PAN dissection did not significantly influence outcomes, as there was little difference in the incidence of complications (55) or survival prognosis (54) between the two groups. In this study, metastases were histologically detected in 8.5% of patients who underwent D2 plus PAN dissection, and the 5-year overall survival rate was 18.2%. Given that nature of prospective randomized trials, these findings support the notion that some patients with latent pathological PAN metastases can survive for 5 years without dissection. In other words, although extended lymph node dissection may be effective for accurate staging, it does not improve survival.

Another important prospective trial was the aforementioned Dutch trial (36, 57), which compared the therapeutic effects of D1 and D2. This trial is significant in that a famous Japanese gastric surgeon provided guidance on the surgical techniques in the Netherlands. Initially, there was no difference in the 5-year survival rate between the D1 and D2 groups (57); however, after 15 years of follow-up, survival outcomes were better among patients who had undergone D2 than among those who had undergone D1 (36). The initial lack of difference in survival was attributed to the relatively greater invasiveness and apparently higher surgery-related mortality of D2 than D1. This study exposed the difficulties of prospective trials of surgical treatment in terms of quality control and evaluation methods. Nevertheless, it was important for demonstrating the superiority of D2 over D1 in patients with advanced gastric cancer. When the results of the JCOG9501 and Dutch trials are integrated, they demonstrate that D2 can improve the prognosis for advanced gastric cancer but that D2 plus PAN dissection has no effect. Furthermore, the integrated results indicate that Asian patients can tolerate the invasiveness of lymph node dissection while Western patients not.

After the clear survival advantages of adjuvant chemotherapy and adjuvant chemoradiotherapy for gastric cancer, prospective studies on lymph node dissection have become less common. Therefore, even though there is a consensus that appropriate prophylactic lymph node dissection improves prognosis, there is not enough evidence to determine the appropriate extent of dissection. The appropriate dissection range for gastric cancer in Western patients and patients with early gastric cancer, obesity, or comorbidities remains to be determined, and the superiority of D2 over D1+ remains unresolved. Currently, the range of D2 is specified for each gastrectomy method in the Japanese guidelines (58, 59), but the validity of each is unknown because no prospective studies have been conducted.

## Challenges in the Preoperative Diagnosis of Lymph Node Metastasis in Gastric Cancer

Preoperative diagnosis of lymph node metastasis is essential for planning proper surgical treatment. However, it is difficult (60), and two factors make the preoperative diagnosis of nodal metastasis difficult: (a) The regional lymph nodes are in the abdominal cavity, and (b) approximately half of lymph node metastases are microscopic (61, 62). According to a recent study on diagnostic imaging (61), which classified metastatic lymph nodes based on microscopic metastatic morphology, 42.5% of metastatic lymph nodes in gastric cancer were of the small nodular type and peripheral type. Lesion volumes for these types are smaller than those for the large nodular and diffuse types.

Researchers have also examined the significance of ultrasonography for diagnosing lymph node metastasis; however, several studies have demonstrated that gastric cancer ultrasonography does not play a major role in diagnosing lymph node metastasis (60, 63, 64). The most important regional lymph nodes in breast cancer are the axillary lymph nodes, which are located under the skin, making ultrasonographic diagnosis easy and ultrasound-guided needle biopsy possible (65–67). On the other hand, since the regional lymph nodes of gastric cancer are in the abdominal cavity, it is difficult to make an ultrasonographic diagnosis from the body surface unless the patient has advanced metastasis, and needle biopsy is not possible. Endoscopic ultrasonography allows for observation within the lumen of the stomach and is more useful than ultrasonography on the body surface (68–70). In esophageal cancer, many of the regional lymph nodes are within the mediastinum around the esophagus; therefore, endoscopic ultrasonography, endoscopic ultrasound-guided elastography, and ultrasound-guided needle biopsy are extremely useful for preoperative diagnosis of lymph node metastasis (70–73). On the other hand, in gastric cancer, although most of the regional lymph nodes are located near the stomach, they lie within the perigastric mesentery (e.g., omentum), and the arteriovenous system runs in the immediate vicinity. Thus, even with endoscopic ultrasonography, it is difficult to visualize regional lymph nodes using ultrasound-guided needle biopsy (60, 74).

Computed tomography (CT) is the most important tool for preoperative nodal diagnosis of gastric cancer (60), and its diagnostic accuracy has increased with technological improvements in equipment (75). Currently, multi-detector spiral CT (MDCT) is widely used (75–82). The advantages of MDCT include objective anatomical imaging and superior spatial resolution. At present, with three-dimensional imaging and multi-planar reconstruction technology, it is possible to determine the precise position and shape of lymph nodes in all directions using different sections. However, even with MDCT, the preoperative nodal diagnosis of gastric cancer is not always satisfactory (81, 82). In a multicenter study concerning preoperative diagnosis of stage III gastric cancer, the sensitivity and specificity of the diagnostic method were 62.5% and 65.7%, respectively (81). A standard nodal diagnosis by MDCT is made based on the assumption that the size of the metastatic lymph node is large (60, 61, 75–82). However, metastatic nodes are not necessarily large (61, 62). In addition, the increased resolution does not mean that all regional lymph nodes can be visualized (61), and accurate calculation of diagnostic ability is difficult given the difficulty in achieving one-to-one correspondence between imaging and pathological diagnosis. In a recent article, the threshold from the receiver operating characteristic (ROC) curve was 7.6 mm in the long axis, and the sensitivity and specificity of the diagnostic method were 86.8% and 80.1%, respectively. However, after one-to-one correspondence based on accurate mapping and measurement of nodal size on resected specimens, the sensitivity and specificity were 91.4% and 47.3%, respectively, even when using the same threshold (61). The reason for such high sensitivity is that large nodes are often metastatic, while the low specificity can be explained by the fact that many metastatic nodes in gastric cancer are small. In this study, 56.3% of the metastatic nodes were below the threshold (61). Thus, patients diagnosed as positive for metastasis using MDCT are extremely likely to be positive for metastasis, but it is difficult to make a definitive diagnosis in node-negative patients. In other words, although MDCT diagnosis is beneficial for advanced gastric cancer, it is not suitable for confirming node-negative early gastric cancer (61, 82). One meta-analysis has indicated that the diagnostic ability of MDCT for lymph node metastasis of gastric cancer was greater for cases with exposed serosa than for cases without serosa exposure (75).

Positron emission tomography-CT (PET-CT) and magnetic resonance imaging (MRI) have also been used for preoperative imaging of lymph node metastasis (60, 80, 83–88), although neither has surpassed the diagnostic ability of MDCT (60). It is difficult to visualize microscopic metastases, even with PET-CT (83, 84). Although attempts have been made to diagnose nodal metastasis with MR lymphography using an ultrasmall superparamagnetic iron oxide (USPIO) contrast medium (88), no sufficient diagnostic results have been reported.

Some articles have reported attempts to improve the diagnostic imaging accuracy for nodal metastasis in gastric cancer. Since diagnostic imaging can determine not only the presence and size of lymph nodes but also the morphology, attempts have been made to improve its accuracy in diagnosing metastasis by taking these factors into consideration. EUS not only identifies perigastric lymph nodes, it also visualizes some internal structure of the lymph nodes, which can sometimes lead to the detection of the intranodal metastatic lesions (89–91). In MDCT, attempts are being made to recognize metastasis from the aspect ratio of lymph nodes and contrast pattern (60, 61, 76). Since the sizes of the regional lymph nodes in gastric cancer vary depending on the lymph node stations, attempt have been reported to set different threshold values for each station, without making it uniform (79).

## An Attempt to Predict Lymph Node Metastasis in Early Gastric Cancer by Using Nomogram and Molecular Makers

There is a limit to diagnosing the presence of lymph node metastasis on diagnostic imaging for gastric cancer. However, if the presence of lymph node metastasis can be inferred from the state of the primary lesion, it may be possible to compensate for the uncertainty in diagnostic imaging. The rate of lymph node metastasis in advanced gastric cancer is high, and the degree of lymph node metastasis is of more importance than the presence or absence of lymph node metastasis. Therefore, it is more important to predict the presence or absence of lymph node metastasis in early gastric cancer than in advanced gastric cancer.

Attempts to calculate regression equations or nomograms for diagnosing the possibility of lymph node metastasis from the clinicopathological factors of early gastric cancer have been reported (92–95). However, unlike extracting the conditions for node-negative patients, attempts to diagnose node-positive patients are not always successful. This type of study generally analyzes patients (who have undergone surgical resection) based on tumor size, site of occupation, histology, depth of invasion, and lymphovascular invasion in resected specimens, but since many of these factors are known after resection, it is difficult to pin-point patients with metastases using only the factors available before surgery.

In order to solve this problem, an attempt to predict the presence of lymph node metastasis by adding molecular markers to clinicopathological factors has been reported. Microarray analysis has led to the observation of gene expressions involved in invasion and metastasis. In gastric cancer as well, upregulation and downregulation of many genes have been observed in relation to lymph node metastasis in basic studies (96–98). However, few molecular markers have proven useful in diagnosing lymph node metastasis in actual clinical specimens. These include VEGF-C (43, 44), EGFR (99), E-cadherin (100, 101), CD44v6 (102), and p53 (103, 104). Unfortunately, these are still in the research stage and have not yet been used in clinical practice. A reason for this is that the usefulness of these assays varies depending on the researcher (102); the heterogeneity of the expression site of the molecular marker may be another reason. In addition, a weak reason is that this is an indirect diagnosis, which does not directly diagnose lymph node metastasis.

## Intraoperative Diagnosis of Lymph Node Metastasis in Gastric Cancer

Researchers have investigated the accuracy of evaluating lymph node metastasis in early gastric cancer *via* sentinel lymph node biopsy (105–108). A sentinel node is defined as a node that directly receives lymphatic drainage from a primary tumor (109). The results of a multicenter prospective study indicated that the sentinel node concept is also valid for early gastric cancer (106). The subject of sentinel node biopsy is a clinical node-negative patient, and although the spread of metastasis is unknown, sentinel node biopsy exhibits excellent performance, making it complementary to MDCT diagnosis (18, 110).

Unfortunately, sentinel node biopsy cannot overcome all the disadvantages of MDCT. First, sentinel node biopsy is beneficial only for patients within the indication, as it is feasible only for cT1N0 gastric cancer of less than 5 cm in size. There are also problems specific to gastric cancer sentinel node biopsy, including the use of lymphatic basin dissection as the standard method, which results in the need for dissection of some regional nodes. Therefore, unlike in cases of breast cancer, sentinel node biopsy is difficult to perform prior to gastrectomy (110). Another disadvantage is that it is technically difficult and requires extensive medical resources (18).

As with other carcinomas, sentinel node biopsy for gastric cancer is advantageous in its capacity for ultra-staging and omitting unnecessary nodal dissection in node-negative patients. As with axillary dissection in patients with breast cancer, researchers have investigated the value of sentinel node biopsy as an indicator for the application of function-preserving curative gastrectomy, which omits nodal dissection and reduces the extent of gastrectomy in patients with gastric cancer. A prospective clinical trial is currently ongoing (108, 111).

## Challenges in the Pathological Investigation of Lymph Node Metastasis in Gastric Cancer

Although it is not often mentioned, there are some pitfalls in the pathological determination of lymph node metastases in gastric cancer, including the accuracy of the number of lymph nodes to be examined and the physical limitations in determining pathological metastasis.

In the old Japanese Classification of Gastric Carcinoma (112), the N stage was determined based on the location of lymph node metastasis. At this time, staging was possible when the presence or absence of metastasis of the most distal lymph node was known and harvesting all dissected lymph nodes was not always necessary. However, with this method, an accurate N stage cannot be determined until a certain extent of lymph node dissection is performed. Currently, the N stage is determined based on the number of metastatic lymph nodes (2, 3). This method is useful for generalization because it does not require complicated grouping or extended nodal dissection. However, all dissected lymph nodes must now be sent to pathology for staging, making it necessary to harvest all lymph nodes removed.

Gastric cancer has many regional lymph nodes, many of which are small, and metastases can occur even in these small lymph nodes (61, 62), which makes harvesting the nodes difficult. However, until now, the accuracy of node harvesting has been neglected (113, 114). Worldwide, harvested nodes are likely to be handled by pathologists. The “palpitation method” for discriminating lymph nodes is probably the most practiced method worldwide and can be performed by pathologists (113), but the number of lymph nodes is larger when examined by surgeons or at a specialized facility (115–117). In clinical practice, more than 15 lymph nodes are often targeted for harvesting (118). However, the actual number of affected lymph nodes is higher, and previous studies have reported that the number of lymph nodes harvested after D2 distal gastrectomy can exceed 40 (113, 119, 120). Many reports have suggested that a greater number of harvested lymph nodes is associated with better prognosis (121–123). In other words, although lymph node harvesting is an important prognostic factor, quality control remains inadequate. The packet submission method has been proposed as a strategy for improving accuracy (115, 124, 125). Although the detection accuracy of the fat-cleaning method has also been reported (126), the time and effort required for fixation, dyeing, and harvesting are extensive.

There are also physical limitations to pathological determination of nodal metastasis. In general, lymph node metastasis is determined *via* microscopic examination of the largest section containing the hilus after hematoxylin and eosin (H&E) staining (2, 3). However, this method can only detect metastatic lesions in the section and may not detect metastasis in the initial image. Lymph node metastasis is thought to progress from cancer cells that flow from the primary lesion into the lymphatic system and reach the marginal sinuses of nodes (110), and the initial images reflect isolated tumor cells (ITCs) and micrometastases. The IJCC staging manual defines an ITC as a metastatic lesion of 0.2 mm or less and a micrometastasis as a metastatic lesion of 2 mm or less (1). Although ITCs do not affect the prognosis of gastric cancer (127), micrometastasis is often reported to worsen the prognosis (128, 129). In other words, when metastasis is determined *via* microscopic examination of the H&E-stained section as usual, a certain degree of error must be considered when determining the number of metastases. For accurate detection of all micrometastases, all retrieved lymph nodes should be subjected to multiple sectioning at 2-mm intervals, which is not practical. An alternative to multiple sectioning is molecular diagnosis of homogenized whole lymph nodes (129). However, there are also challenges to overcome in molecular diagnosis, such as the selection of the gene amplification method, primer selection, contamination, pseudogenes, and cost, making it impractical for use in actual clinical practice. Furthermore, how the results of molecular diagnosis should be used to determine prognosis remains unknown.

## Current State and Future Developments in the Evaluation of Lymph Node Metastasis in Gastric Cancer

To a certain extent, nodal metastasis of gastric cancer can be cured *via* lymph node dissection when limited to the perigastric nodes. Therefore, preoperative nodal diagnosis is important for planning surgical treatment in patients with gastric cancer. In the case of advanced gastric cancer, MDCT can be used for preoperative nodal diagnosis to some extent, although it is difficult to distinguish node-negative patients with early gastric cancer using this method. Sentinel node biopsy can overcome some of the disadvantages of MDCT.

Postoperative nodal metastatic status is also important when determining the strategy for adjuvant chemotherapy after gastrectomy. However, since the degree of nodal metastasis is determined based on the number of metastatic nodes, it should be noted that there are potential problems with the accuracy of harvesting and a possibility of underestimating micrometastasis. A practical solution to this problem would be to add a safety margin when performing lymph node dissection in patients undergoing gastrectomy.

In the remainder future developments surrounding lymph node metastasis in gastric cancer will be discussed.

Advancements in CT equipment and diagnosis are expected to continue. Improvements in artificial intelligence (AI) supported diagnosis will likely increase the accuracy of nodal diagnosis, rather than finer resolution and more detailed 3D construction (130–132). Although lymph node morphology and contrast patterns have been useful for nodal diagnosis (133), AI diagnosis is likely to surpass this. Advances in intraoperative nodal diagnosis are also expected. Indeed, recent studies have attempted to detect tumor antigens, enzymes produced by tumors, or stromal reactions surrounding metastases using fluorescence observation for rapid intraoperative diagnosis of metastasis (134–140).

Controlling the accuracy of harvesting remains critical, therefore, thorough analysis of the associated difficulties are required to develop simple standardized methods with better accuracy and objectivity than the palpitation method. The most promising method is the fat-dissociation method (113), which has been reported to be useful for shortening the time and improving the accuracy of node harvesting in patients with gastric cancer. Other methods such as indocyanine green fluorescence and methylene blue staining methods have also been proposed (141, 142).

In addition, therapeutic strategies targeting lymph node metastases, especially sentinel lymph node metastases in the case of molecular targeting therapy, have been considered (143). If such techniques prove useful, the significance of lymph node metastasis will extend beyond a mere basis for staging, and it will become an essential factor when planning more effective adjuvant therapy and treatment strategies for recurrence.

Chemotherapy for advanced gastric cancer has also progressed, and conversion gastrectomy can be performed after downstaging with chemotherapy, even in patients who are not eligible for radical resection (144, 145). It is important to diagnose nodal metastasis and determine its influence on therapeutic efficacy in such patients. To further enhance the effect of conversion gastrectomy following chemotherapy, prompt judgments of diagnosis and the chemotherapeutic effect are essential. Currently, PET-CT is useful; however, there are expectations for improved CT diagnosis using AI.

## Author Contributions

SK was responsible for the scientific conception of the study and writing of the manuscript. All authors (SK, HS, and HT) contributed to the literature review, data analysis, drafting, editing, critical revision of the manuscript, and approval of the final version of the manuscript.

## Conflict of Interest

The authors declare that the research was conducted in the absence of any commercial or financial relationships that could be construed as a potential conflict of interest.

## Publisher’s Note

All claims expressed in this article are solely those of the authors and do not necessarily represent those of their affiliated organizations, or those of the publisher, the editors and the reviewers. Any product that may be evaluated in this article, or claim that may be made by its manufacturer, is not guaranteed or endorsed by the publisher.
